# O04 UKHSA Start Smart Then Focus antimicrobial stewardship: effective implementation during the COVID-19 pandemic at an NHS Foundation Trust in the UK

**DOI:** 10.1093/jacamr/dlae136.004

**Published:** 2024-08-23

**Authors:** Rasha Abdelsalam Elshenawy, Nkiruka Umaru, Zoe Aslanpour

**Affiliations:** Department of Pharmacy, School of Life and Medical Sciences, University of Hertfordshire, Hatfield AL10 9AB, UK; Department of Pharmacy, School of Life and Medical Sciences, University of Hertfordshire, Hatfield AL10 9AB, UK; Department of Pharmacy, School of Life and Medical Sciences, University of Hertfordshire, Hatfield AL10 9AB, UK

## Abstract

**Background:**

Antimicrobial resistance (AMR), a major global public health threat that has caused 1.2 million deaths, calls for immediate action. Antimicrobial stewardship (AMS) promotes judicious antibiotic use, but the COVID-19 pandemic increased AMR by 15%.^1^

**Objectives:**

To investigate the AMS implementation prior to and during the COVID-19 pandemic.

**Methods:**

This cross-sectional retrospective study was undertaken to estimate the prevalence of inappropriate antibiotic prescribing, focusing on adult patients treated for respiratory tract infections (RTIs). It investigates antibiotic prescribing practices for RTI conditions such as pneumonia and COVID-19-related RTIs in 2020. A total of 640 patient records, 320 in each year, were retrospectively analysed using the UK Health Security Agency’s (UKHSA) ‘Start Smart, Then Focus’ (SSTF) AMS toolkit at one NHS Foundation Trust.^2^ Ethical approval was secured and public and patient involvement through the Citizens Senate was integral, with registration in ISRCTN related to WHO criteria and Octopus.

**Results:**

During the pandemic, the ‘Start Smart’ antibiotic approach involved a retrospective analysis of 640 patient records from a Trust, showing stable prescription appropriateness with a marginal age increase from an average of 74.3 to 76.2 years (*P*=0.127). The predominant age group remained 66–85 years, with a slight decrease from 48.8% to 46.3%. The length of hospital stay decreased from 13.7% to 12.3% on average. Mortality rates were constant at 15%. Notably, ‘side effects’ as an antibiotic allergy classification changed significantly (*P*=0.023). Community-acquired pneumonia was the primary diagnosis, with uncertain admission diagnoses influencing antibiotic choice. Significant comorbidity shifts were noted; heart failure increased (*P*=0.007) while kidney diseases decreased (*P*=0.008). Antimicrobial stewardship interventions showed a notable rise in ‘De-escalation’ (*P*=0.005), with guideline adherence dropping from 64% to 36% during the pandemic (Figure 1).

**Conclusions:**

The study highlights how AMS practices, such as ‘De-escalation’, have been pivotal in antimicrobial management during the pandemic. The resilience of AMS in this crisis indicates that sustainable, adaptable AMS measures are essential in the post-pandemic era to continue saving lives. The impact of this research study is significant, offering insights that enrich the global conversation on antimicrobial stewardship. It delves into the core aspects of AMS, emphasizing its vital role in everyday healthcare and its increased importance during public health crises, demonstrating the potential to save lives by reducing antimicrobial resistance.

**Figure 1.**
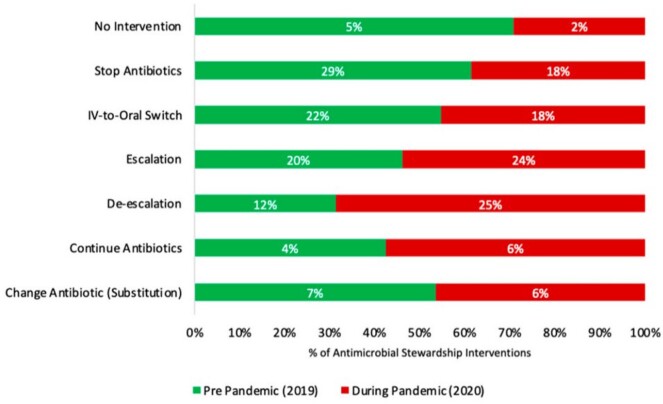
Antimicrobial stewardship interventions prior to and during the COVID-19 pandemic.
